# 

**Published:** 1896-05-09

**Authors:** 


					J -llUJj [.UUJi n JTO. ALtUUtiU X'JUU 1 1 UllLI Lliuiuri
CADBURY'S ww w CADBURY'S
cocoa 1=1 COCOA
"The standard of highest purity at present fcSa f*l ALKALIES USED,
attainable."?Lancet.
<R IYICH HOSft.TAl
No. 502.\fVoi. XX.
8ATUBDAY,
May 9tii, 1896.
WITH EXTRA NURSING SUPPLEMENT.
MMKtodom^d'SS *" gS^.y,K?.i.S,?o?W

				

